# Research of Low-Temperature Performance of Polyphosphoric Acid-Modified Asphalt

**DOI:** 10.3390/ma16010111

**Published:** 2022-12-22

**Authors:** Jianguo Wei, Meiyan Huang, Yuming Zhou, Ping Li, Fan Yu, Haolong Ju, Song Shi

**Affiliations:** 1 School of Traffic and Transportation Engineering, Changsha University of Science and Technology, Changsha 410114, China; 2 National Engineering Research Center of Highway Maintenance Technology, Changsha University of Science and Technology, Changsha 410004, China; 3 Department of Municipal and Road and Bridge Engineering, Hunan Urban Construction College, Xiangtan 411101, China; 4 Henan Railway Construction & Investment Group Co., Ltd., Zhengzhou 450018, China

**Keywords:** polyphosphoric acid, low-temperature performance, multi-indicator evaluation, burgers model, modification mechanism

## Abstract

Polyphosphoric acid (PPA) modifier, which can effectively improve the rheological properties of asphalt, is widely used in pavement engineering. In order to accurately evaluate the low-temperature performance of PPA-modified asphalt, in this study, PPA-modified asphalt and PPA/SBR-modified asphalt were prepared. The modification mechanism was explored by scanning electron microscopy (SEM) and fourier transform infrared spectroscopy (FTIR). Bending Beam Rheology (BBR) test was carried out, and four indexes, including K index, viscous flow (η_1_), low-temperature integrated flexibility (Jc), and relaxation time (λ), were obtained by combining the Burgers model. The optimal low-temperature performance evaluation index of modified asphalt was determined by the analytic hierarchy process (AHP). The test results show that PPA addition to asphalt will produce chemical reactions, which can effectively improve the compatibility between SBR and neat asphalt. In the multi-index evaluation based on K, η_1_, Jc, and λ, the same optimum content of PPA was obtained. AHP analysis further demonstrates that Jc is the optimal evaluation index for laboratory research on the low-temperature performance of PPA-modified asphalt, and λ index is the ideal evaluation index for the low-temperature performance of asphalt in engineering applications.

## 1. Introduction

Asphalt pavement has become a preferred choice for high-grade paved surfaces because of its advantages. For example, it is smooth, it is fast to construct and does not require curing time, and it is easy to maintain and repair. However, the traffic volume is growing rapidly, and vehicle overload and road channelization are getting worse. Subsequently, problems like potholes, ruts, and cracks start to appear in asphalt pavements that have been only used for 7 to 10 years. These problems lead to rainwater infiltration, reducing the service life of asphalt pavement. Modifier addition is a common method to improve the performance of base asphalt [1,2].

Current asphalt modifiers mainly include thermoplastic elastomers (e.g., styrene-butadiene-styrene block copolymer, SBS), natural and synthetic rubbers (e.g., styrene-butadiene rubber, SBR), thermosetting materials (e.g., epoxy resins), asphalt chemical modifiers (e.g., polyphosphoric acid, PPA), and warm mix modifiers [1,2]. Among them, SBR is considered the most effective one, as SBR addition can improve the high- and low-temperature performance of neat asphalt and enhance its adhesion and deformation ability [2,3]. This is mainly because SBR modifier constructs a cross-linked three-dimensional network structure in asphalt. However, SBR-modified asphalt is susceptible to oxidation and aging on account of a large amount of butadiene structure in the SBR molecule [4]. In addition, the compatibility and storage stability of SBR-modified asphalt is poor, and the cost price is relatively high [5]. On this basis, researchers have attempted to improve compatibility and storage stability using physical or chemical methods in combination with other modifiers.

PPA is a chemical modifier consisting of orthophosphoric acid, various polymeric polyphosphoric acids, and some metaphosphoric acids. It is favored by road engineers because of its good compatibility with asphalt, excellent thermal storage stability, and low price. The mechanism of asphalt modification by PPA depends on the type of neat asphalt. Because the chemical composition of asphalt from different origins varies greatly, different conclusions on the modification mechanism of PPA-modified asphalt have been drawn. Researchers have put forward a more reasonable explanation from the perspective of the interaction between PPA and asphalt components [6,7,8]: Large amounts of H_2_PO_4_^−^ and H^+^ can be produced after adding PPA to asphalt, which induces protonation reactions, leading to the loss of hydrogen bonds and asphaltene dispersion in asphaltene. A stable spatial network structure can be formed by the dispersed asphaltene units, which makes the asphalt more elastic. In addition, the alkyl aromatic hydrocarbons in the resin are decomposed into aromatic compounds insoluble in n-heptane, which are precipitated as asphaltenes, resulting in a decrease in the content of the resin and an increase in the content of asphaltenes; these changes indicate the improvement in the macroscopic high-temperature performance of asphalt at the macro-level.

Researchers have reached a consensus that PPA can effectively improve the high-temperature stability and fatigue resistance of asphalt [7,9], and when PPA is combined with polymer modifiers such as SBS and SBR, it can effectively improve the compatibility and storage stability of polymer-modified asphalt [10,11,12]. However, no agreement has been reached on the low-temperature performance of PPA-modified asphalt and its evaluation method [13]. Zhou et al. [14] investigated the low-temperature performance of PPA-modified asphalt with different asphalt matrices and found that the modification of asphalt binder by PPA was related to the chemical composition of asphalt and that PPA degraded the low-temperature performance of Esso-70#. Similar conclusions were also reached by Aflaki [15] and Sarnowski [8]. Wei et al. [10], Zhang et al. [16], and Liu et al. [17] found that PPA affected the ductility of the neat asphalt based on conventional ductility tests and rheological performance tests. The low-temperature properties of the asphalt modified by PPA and SBS/rubber were poor. Some studies also demonstrated that low-temperature properties of asphalt could be enhanced by PPA. Baldino et al. [18] and Wang et al. [19] found that the low-temperature crack resistance of asphalt could be improved by adding PPA, and the improvement was positively correlated with the PPA dosage. Baldino et al. [13], Thomas et al. [20], and Edwards et al. [21] used glass transition temperature and brittle point as evaluation indexes. They found that after PPA addition, the temperature when asphalt turned to a glassy state was effectively reduced; the elasticity of asphalt was indirectly increased, and its low-temperature crack resistance was enhanced.

There is no evaluation method for PPA-modified asphalt and related composite modified asphalt in current technical specifications in China. The low-temperature evaluation indicators also need to be perfected. Currently, the main indicators for the low-temperature evaluation of PPA-modified asphalt include the glass transition temperature, low-temperature needle penetration, ductility, brittle point, creep stiffness modulus (S), and creep rate (m) [10,13,19]. However, glass transition temperature, low-temperature needle penetration, and ductility are less relevant to the actual low-temperature road properties of the mixtures [22,23]. Additionally, the evaluation by the creep stiffness modulus (S) or creep rate (m) alone has limitations. Theoretically, the improvement in the low-temperature performance of asphalt is indicated by the decrease in the S value and the increase in the m value, but the situation where the S value and the m value increase or decrease at the same time may occur [24]. In this situation, the low-temperature performance of asphalt cannot be directly evaluated. S represents the low-temperature deformation capacity of asphalt, and m represents the low-temperature stress relaxation capacity of asphalt, and they cannot be comprehensively considered.

**Figure 1:**
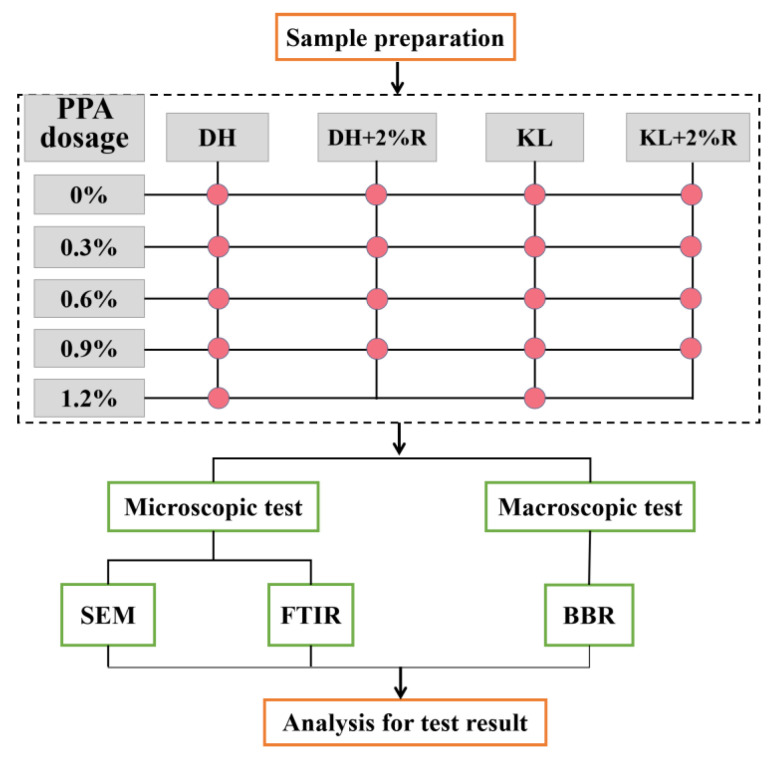
Experimental diagram of this study.

In summary, for determining an ideal indicator for the low-temperature performance of asphalt, the low-temperature deformation capacity and low-temperature stress relaxation capacity should both be considered. In this paper, scanning electron microscopy (SEM) tests were performed to characterize the compatibility of the modified asphalt. In addition, fourier transform infrared spectroscopy (FTIR) tests are carried out to characterize the modification mechanism. Based on viscoelastic theory and bending beam rheometer (BBR) test, the K index, and Burgers model-based viscous flow (η_1_), low temperature integrated flexibility (Jc), and relaxation time (λ) were used to comprehensively evaluate the low-temperature performance of modified asphalt. Finally, the optimal low-temperature performance evaluation indicator is determined by the analytic hierarchy process (AHP), The experimental diagram of PPA-modified asphalt is shown in Figure 1, which can guide low-temperature performance evaluation and the promotion of PPA-modified asphalt.

## 2. Materials and Methods

### 2.1. Materials

Neat asphalt was Kunlun-90# (hereafter also KL) produced by Qinhuangdao PetroChina Fuel Asphalt Company(Qinhuangdao, China), and Donghai-70# (hereafter also DH) produced by Sinopec (Beijing, China). The technical indexes of asphalt were tested according to the relevant methods in the “Standard Test Methods of Bitumen and Bituminous Mixtures for Highway Engineering” (JTG E20-2011). The results are listed in Table 1. In PPA-modified asphalt, the mass fraction of the PPA (H_3_PO_4_ content of 110%) was 0.3%, 0.6%, 0.9%, and 1.2%. The SBR used in this study was produced by Beijing Yuda (Beijing, China). In the PPA/SBR-modified asphalt, the mass fraction of SBR was 2%, and SBR was compounded with 0.3%, 0.6%, and 0.9% PPA, respectively. The relevant technical indicators are listed in Table 1, Table 2 and Table 3. For the convenience of subsequent analysis, the addition of 0.3% PPA to Kunlun-90# asphalt is expressed as KL + 0.3% P, the addition of 2% SBR to Kunlun-90# asphalt is expressed as KL + 2%, and the addition of 0.3% PPA and 2% SBR to Kunlun-90# asphalt is expressed as KL + 2% R + 0.3% P. All other modified asphalts are represented in this way.

### 2.2. Sample Preparation

**Figure 2:**
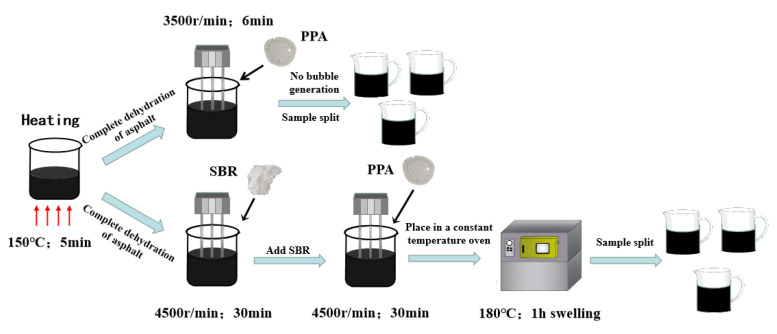
The preparation process for PPA-modified asphalt and PPA/SBR-modified asphalt.

By combining the preparation methods of PPA-modified asphalt used by former scholars and those applied by the group in preliminary research [10,25], the preparation process of PPA-modified asphalt and PPA/SBR-modified asphalt was determined (Figure 2). Considering asphalt specimens need to be heated several times, the prepared modified asphalt was divided into different stainless-steel containers in advance to ensure the same heating process.

### 2.3. Asphalt Binder Test Method

The SEM tests were performed to observe the surface micromorphology of modified asphalt. The neat asphalt in the flowing state and the prepared modified asphalt were poured into circular specimens of suitable size and thickness. The asphalt was cooled and shaped at room temperature, sprayed with gold, placed inside a scanning electron microscope for evacuation, and observed at a magnification of 1.00 kx.

The FTIR tests were conducted to explore the modification mechanism of asphalt. The resolution was 4 cm^−1^, and the number of scans was 32. The wave number test range of the infrared spectrum of modified asphalt was 400 cm^−1^ to 4000 cm^−1^.

The creep stiffness modulus (S) and creep rate (m) of asphalt were determined using the BBR test to evaluate the low-temperature performance of asphalt. The threshold value was set according to the SHRP process: To ensure the low-temperature crack resistance of asphalt pavement, S should not be greater than 300 MPa, and m should not be less than 0.30 at the design temperature. The test temperature in this study was −12 °C.

## 3. Results and Analysis

### 3.1. Compatibility and Micro-Mechanism of the PPA-Modified Asphalt 

#### 3.1.1. SEM Image Analysis

**Figure 3:**
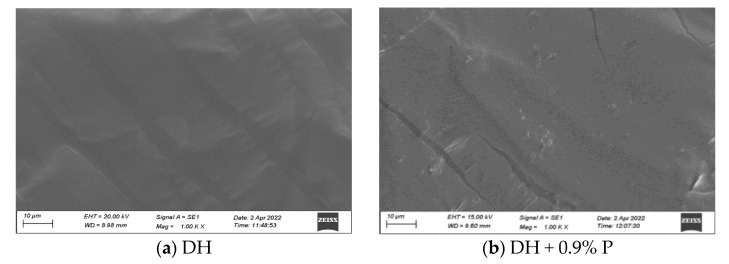

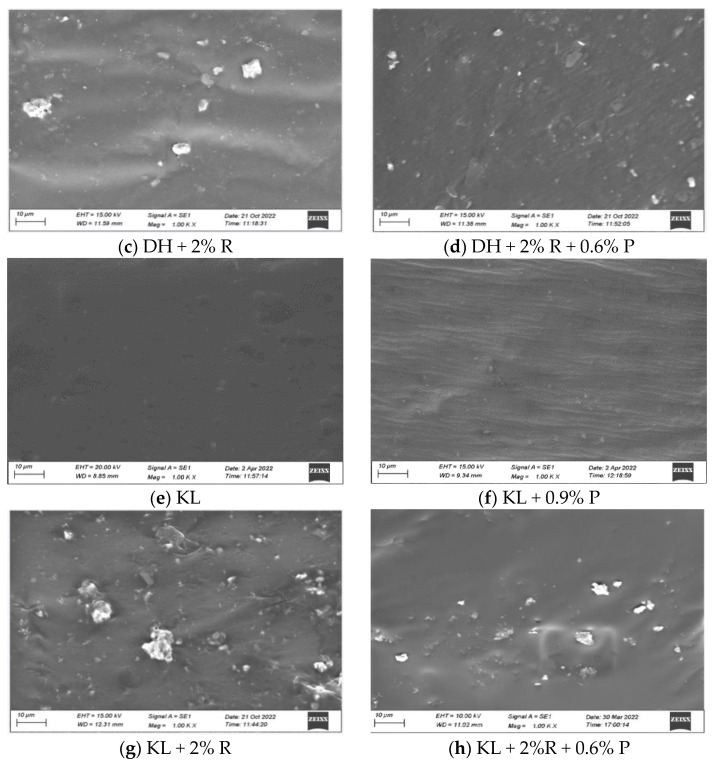
Scanning electron microscopy results of asphalt at 1000 times.

Scanning electron microscope works based on the photoelectron theory and provides a clear and in-depth view of the surface morphology of the sample at the microscopic level. In addition, SEM has an ideal field of view and depth of field, with the advantages of simple sample preparation, less contamination, and rapid detection. It is widely used in various fields, such as materials science, physics, chemistry, and biology [7]. Figure 3 shows the selected specimens for testing, including DH, KL, neat asphalt + 0.9% P, neat asphalt + 2% R, and neat asphalt + 2% R + 0.6% P.

It can be seen that both DH and KL neat asphalts have smooth surfaces without granular or reticulated structures. When 0.9% PPA was added, the surface of the asphalt samples in Figure 3b,f exhibited uniformly distributed microscopic mesh structures in good compatibility with the asphalt. When 2% SBR was incorporated, a larger granular structure occurred on the surface of the asphalt samples in Figure 3c,g, which was less compatible with the asphalt. When 0.6% PPA was blended into 2% SBR-modified asphalt, the granular structure was still observed on the surface of the asphalt samples in Figure 3d,h, but the granular size was reduced, and the degree of agglomeration was decreased compared to the surface of SBR-modified asphalt. This result suggests that the compatibility of SBR in asphalt can be improved by adding PPA. The reason may be that the asphaltene content was increased by incorporating PPA, promoting the asphalt polar force increase, destroying the wax-based structure in asphaltene, and facilitating the compatibility of SBR in asphalt.

#### 3.1.2. Mechanism Analysis of Asphalt Modification

**Figure 4:**
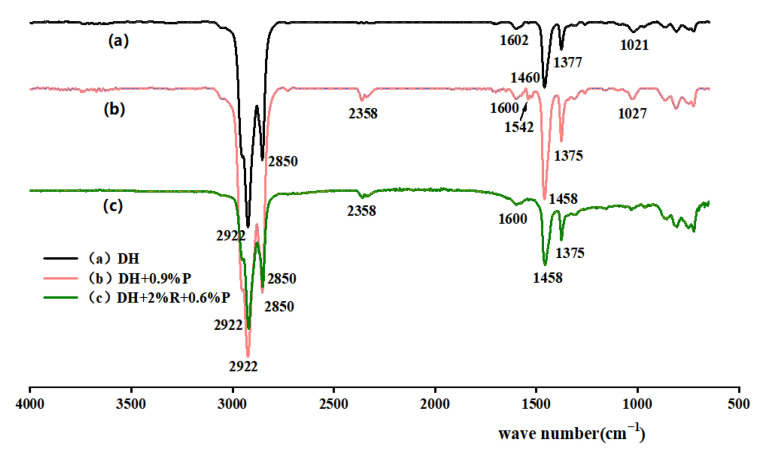
Infrared spectra of DH and its modified asphalts.

**Figure 5:**
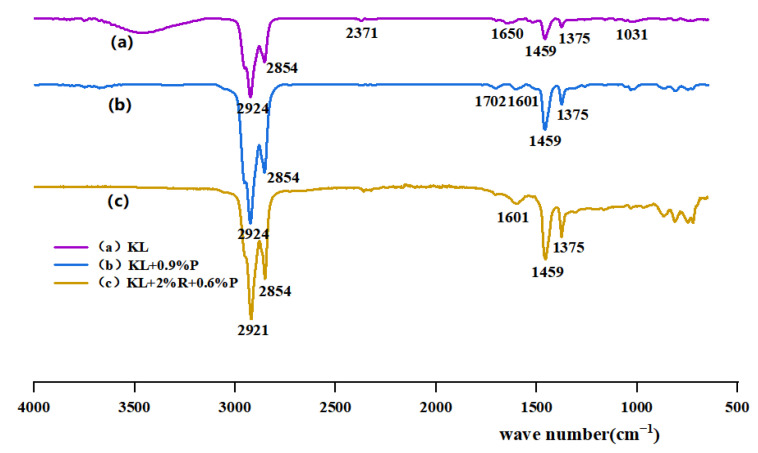
Infrared spectra of KL and its modified asphalts.

An FTIR test was performed to explore the reaction of substances. The characteristic peaks of different compounds and the different wavelengths of absorption light of molecular bond vibrations were analyzed. The obtained infrared spectrogram was divided into the functional group region and the fingerprint region. The wave number of the functional group region is between 1330 cm^−1^ and 4000 cm^−1^, and the region is mainly for group identification and reflects the vibration of characteristic functional groups in each molecule. The wave number in the fingerprint region is between 400 cm^−1^ and 1330 cm^−1^, reflecting small changes in molecular structure and absorbing a complex spectrum. The asphalt specimens of DH, KL, neat asphalt + 0.9% P, and neat asphalt + 2% R + 0.6% P were selected for testing. The results are depicted in Figure 4 and Figure 5.

Figure 4a and Figure 5a show the infrared spectra of the two neat asphalts. There are strong characteristic peaks in the wave number range from 3000 cm^−1^ to 2790 cm^−1^. The antisymmetric stretching vibration peaks of methylene −CH_2_- are at 2922 cm^−1^ and 2924 cm^−1^; the symmetric stretching vibration peaks of methylene −CH_3_- are at 2850 cm^−1^ and 2854 cm^−1^. Due to the symmetric bending vibration of −CH_2_- and the asymmetric bending vibration of −CH_3_-, absorption peaks appear near 1458 cm^−1^ and 1375 cm^−1^, respectively. The absorption peaks at 1021 cm^−1^ in Figure 4a and 1031 cm^−1^ in Figure 5a correspond to the ring vibration of cyclohexane. Figure 4b and Figure 5b show the infrared spectra of PPA-modified asphalt. It has been found that PPA is mainly composed of inorganic phosphates, aliphatic hydrocarbons, and aliphatic amines with other chemical components. The biggest difference between PPA-modified asphalt and neat asphalt is the disappearance and generation of absorption peaks at 1725 cm^−1^–1480 cm^−1^, 2358 cm^−1^ in Figure 4b, and 2387 cm^−1^ in Figure 5b. The main reason is that alcohols in asphalt react with PPA, and the −OH in alcohol is neutralized by phosphoric acid to form phosphate ester [26]. Figure 4c and Figure 5c show the infrared spectra of the composite modified asphalt. The characteristic peaks of both modified asphalt are included in the spectrum, and the disappearance and generation of characteristic peaks are observed near 1600 cm^−1^ in Figure 4c and Figure 5c and 1000 cm^−1^–600 cm^−1^ in Figure 4c, indicating that the chemical modification is caused by the addition of PPA and SBR.

### 3.2. Analysis of Indicators in Low-Temperature BBR Test

#### 3.2.1. Creep Stiffness Modulus (S) and Creep Rate (m)

Creep stiffness modulus (S) and creep rate (m) of PPA-modified asphalt and PPA/SBR-modified asphalt at −12 °C were determined to characterize the deformation capacity and stress relaxation capacity of asphalt at low temperatures. The test results are shown in Table 4 and Table 5. AASHTO [27] concluded that a smaller S leads to a larger m, indicating good low-temperature properties of asphalt; conversely, indicating that asphalt is hard and brittle at low-temperature conditions.

**Figure 6:**
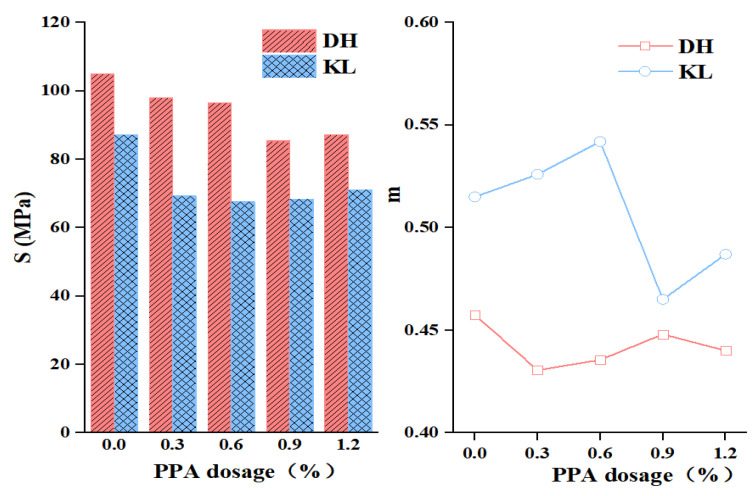
S and m of single modified asphalt with different PPA dosages.

**Figure 7:**
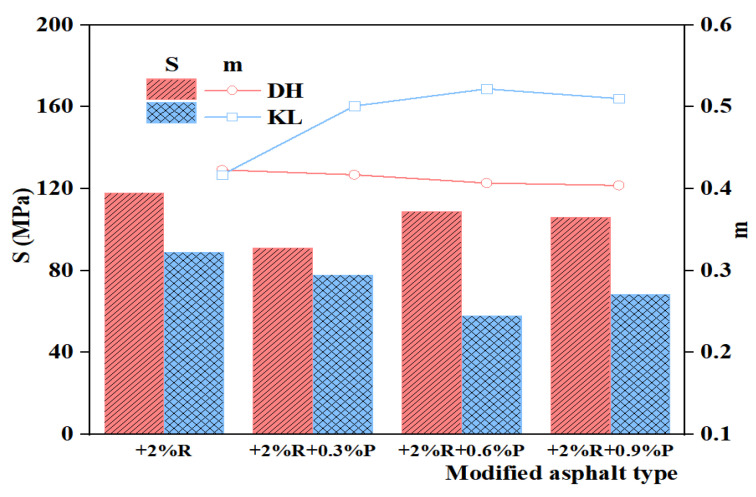
S and m of composite modified asphalt with different PPA dosages.

Figure 6 and Figure 7 reveal that with the increase of PPA dosage, the S of PPA-modified asphalt gradually decreases and m gradually increases, with a turning point at 0.9% admixture. The change indicates that the addition of PPA can improve the low-temperature performance of modified asphalt, and the improvement is related to the PPA dosage. The S of SBR-modified asphalt is negatively correlated with the SBS admixture, but m is positively correlated with the SBS admixture. After adding PPA, the S of PPA/SBR modified asphalt decreases first and then increases, indicating that PPA can replace part of SBR and achieve the same asphalt performance. The variation of m is related to the type of neat asphalt, with a slight decrease for DH composite modified asphalt and an increase for KL composite modified asphalt.

By further analyzing the effect of PPA dosage on the low-temperature performance of asphalt, the evaluation with S or m alone was found to have some limitations. As reflected by the KL-PPA-modified asphalt in Figure 7, S gradually decreases with the increase of PPA dosage, while the corresponding m shows a fluctuating trend rather than a continuous increase. Therefore, it is one-sided to evaluate the low-temperature performance of asphalt by only considering the deformation capacity or stress relaxation capacity.

#### 3.2.2. K Index

**Figure 8:**
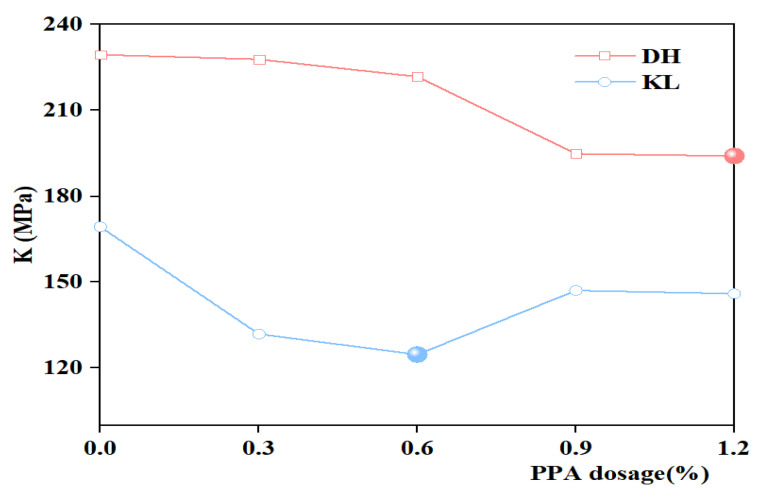
K index of single modified asphalt with different PPA dosages.

**Figure 9:**
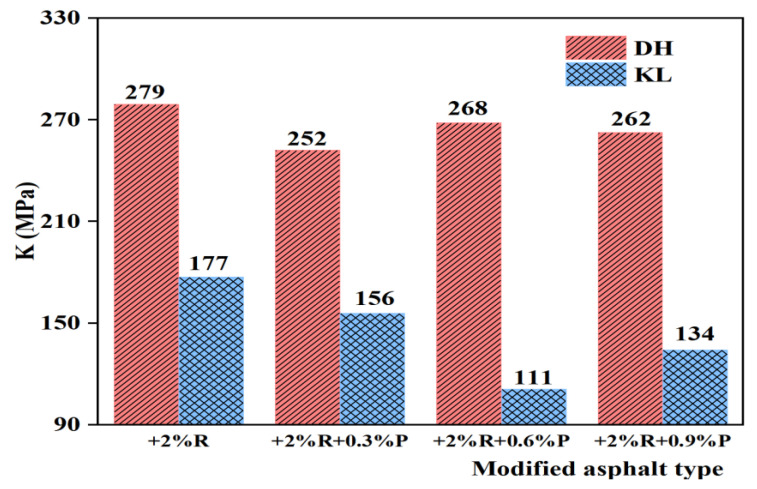
K index of composite modified asphalt with different PPA/SBR dosages.

Since the deformation and stress relaxation capacity of asphalt is not considered when using S or m to evaluate the low-temperature performance of asphalt, Tan et al. [28] found that the K index correlates well with the low-temperature performance of asphalt. It can significantly distinguish the low-temperature performance of different neat asphalt and modified asphalt, as expressed by Equation (1). In addition, the K index and S are positively correlated, with a smaller K index indicating a better low-temperature performance of the asphalt. The creep stiffness modulus (S) and creep rate (m) at 60 s were used as low-temperature evaluation indicators, and the K index of PPA-modified asphalt with different PPA dosages was calculated according to Equation (1). The results are shown in Figure 8 and Figure 9.

(1) $$K=\frac{S}{m}$$

It can be seen from Figure 8 and Figure 9 that K indexes of PPA-modified asphalt and PPA/SBR-modified asphalt are smaller than those of neat asphalt or SBR-modified asphalt, indicating that PPA can improve the low-temperature performance of asphalt. For PPA-modified asphalt, the K index of DH-PPA-modified asphalt decreases as the PPA dosage increases. At the PPA dosages of 0.9% and 1.2%, the K index is similar, and the corresponding modified asphalt has better low-temperature performance. The K index of KL-PPA-modified asphalt first decreases and then increases, with a recommended PPA dosage of 0.6%, indicating that the recommended PPA dosage is related to the type of neat asphalt. For PPA/SBR-modified asphalt, the K index decreases first and then increases with the increasing PPA dosage, and the lowest point appears. Therefore, the recommended dosage of PPA for DH-PPA/SBR-modified asphalt is 0.3%, and that of KL-PPA/SBR-modified asphalt is 0.6%.

#### 3.2.3. Viscous Flow (η_1_)

**Figure 10:**
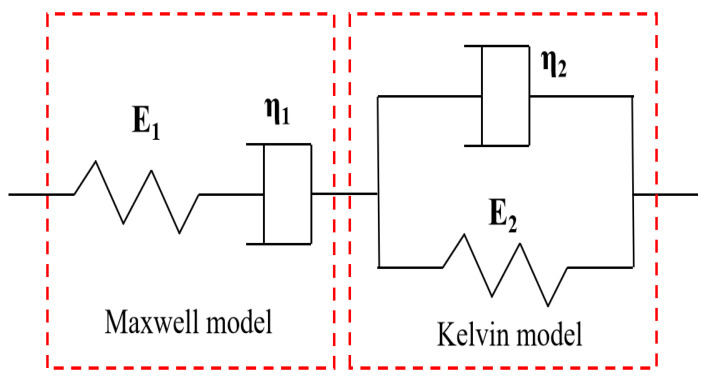
Schematic of the Burgers model with dashpot and spring components.

The composition of asphalt is complex, with viscosity and elasticity. Therefore, the Burgers model is often used to describe the mechanical behavior of asphalt to study its rheological properties. By fitting the Burgers model to the whole process of the asphalt BBR test, the low-temperature creep performance of asphalt can be reflected [29]. The Burgers model combines the advantages of both Maxwell and Kelvin models, and the four-parameter model can be obtained by connecting the two models in series, as shown in Figure 10.

According to the constitutive equation of the Burgers model, the equation of creep flexibility is derived as follows:

(2) $$J(t)=\frac{1}{E_{1}}+\frac{t}{\eta_{1}}+\frac{1}{E_{2}}({1-e}^{-\frac{E_{2}}{\eta_{2}}t})$$

where J(t) is the creep flexibility (MPa); E_1_ denotes the instantaneous modulus of elasticity (MPa); E_2_ is the delayed modulus of elasticity (MPa); η_1_ represents the viscous flow parameter (MPa·s); η_2_ is the delayed viscous flow parameter (MPa·s).

**Figure 11:**
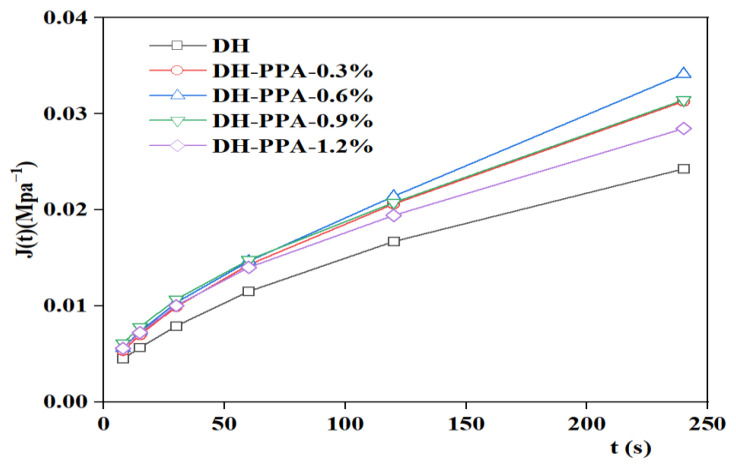
Creep flexibility curve at −12 °C.

**Figure 12:**
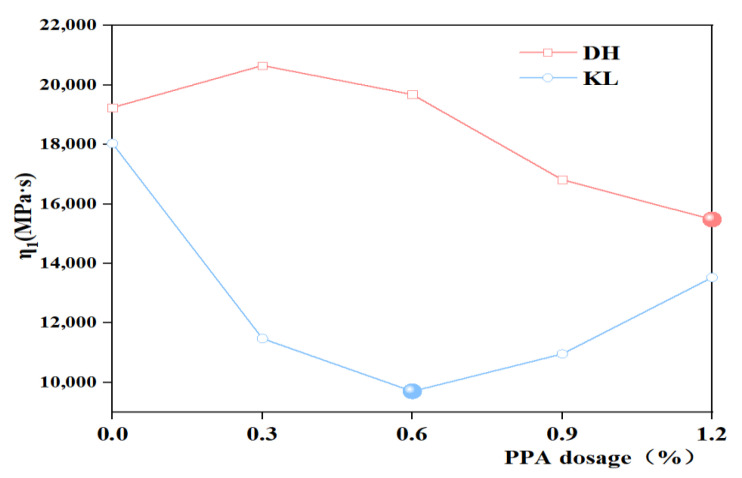
The η_1_ value of single modified asphalt with different PPA dosages.

**Figure 13:**
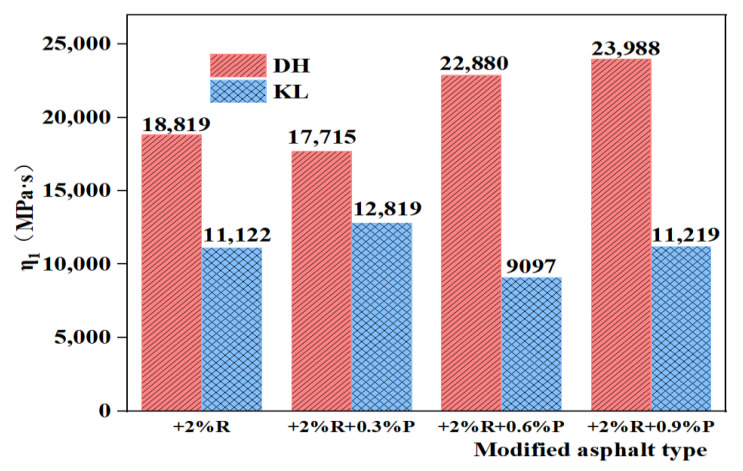
The η_1_ value of composite modified asphalt with different PPA/SBR dosages.

The creep flexibility curve was fitted using the 1stopt nonlinear curve fitting function to derive the four parameters (E_1_, E_2_, η_1_, η_2_) of the Burgers model, and the J(t) values were calculated. The variation of DH and DH-PPA/SBR asphalts is illustrated in Figure 11. It has been found that the viscous flow parameter η_1_ can be used to characterize the low-temperature deformation capacity of asphalt, with a smaller η_1_ indicating a better low-temperature crack resistance of asphalt [30]. Therefore, the viscous flow parameter η_1_ was used as an evaluation indicator to plot the variation of viscous parameters with different PPA dosages (Figure 12 and Figure 13). The analysis of η_1_ variation shows that the recommended PPA dosage is 1.2% for DH-PPA-modified asphalt, 0.6% for KL-PPA-modified asphalt, 0.3% for DH-PPA/SBR-modified asphalt, and 0.6% for KL-PPA/SBR-modified asphalt.

#### 3.2.4. Low Temperature Integrated Flexibility (Jc)

The creep equation of the Burgers model is divided into the instantaneous elastic part (J_E_), the delayed elastic part (J_De_), and the viscous part (J_V_). Their relationship can be expressed by Equation (3) [31]:

(3) $$\left\{ \begin{array}{l} J(t)=J_{E}+J_{De}+J_{V}\\ J_{E}=\frac{1}{E_{1}};J_{De}=\frac{1}{E_{2}}(1-e^{\frac{-tE_{2}}{\eta_{2}}});J_{V}=\frac{t}{\eta_{1}} \end{array}\right.$$

Jc is a comprehensive indicator for evaluating the low-temperature performance of asphalt in the Burgers model and can be calculated by Equation (4). Asphalt removes stress relaxation by flowing due to its viscosity when the temperature is low, thus reducing the probability of asphalt cracking. Therefore, a smaller Jc was obtained when J_v_ became larger, suggesting a stronger low-temperature performance [24].

(4) $$J_{C}=1/[J_{V}\times (1-\frac{J_{E}+J_{De}}{J_{E}+J_{De}+J_{V}})]$$

**Figure 14:**
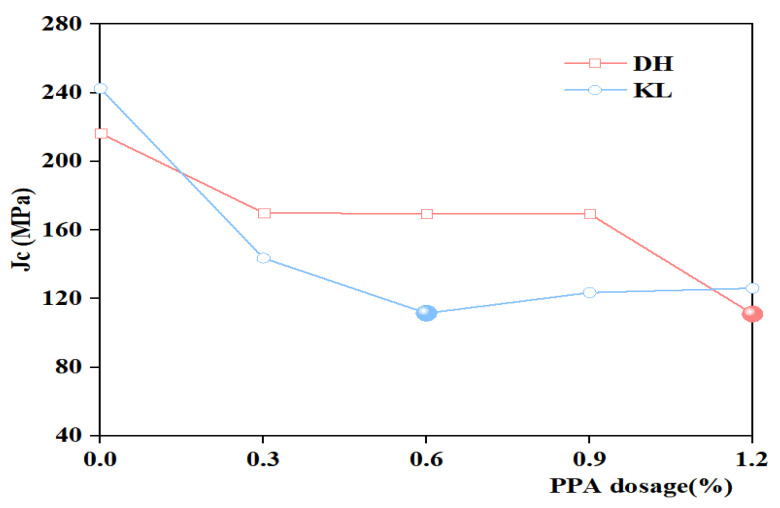
The Jc value of single modified asphalt with different PPA dosages.

**Figure 15:**
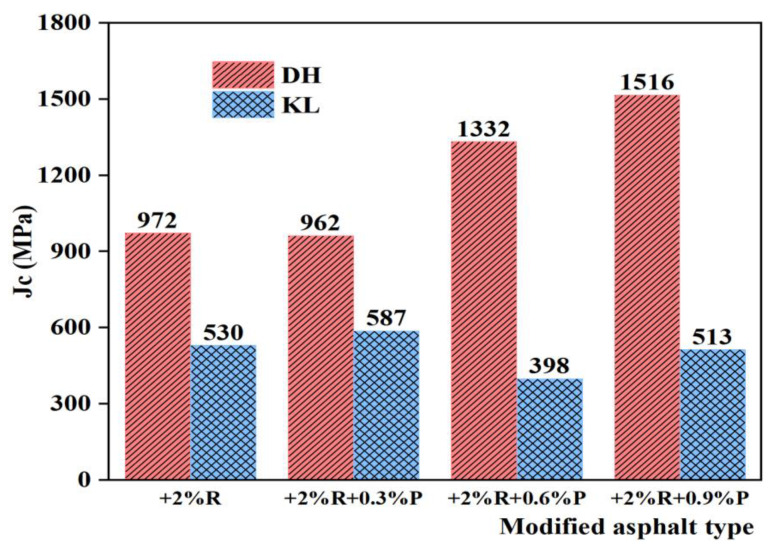
The Jc value of composite modified asphalt with different PPA/SBR dosages.

Figure 14 and Figure 15 show the Jc of PPA-modified asphalt and PPA/SBR-modified asphalt. It can be seen that the Jc of different asphalt types varies with PPA dosages. In the PPA-modified asphalt, Jc decreases with increasing PPA dosages, indicating that PPA can improve the low-temperature performance of asphalt. After adding PPA to SBR-modified asphalt, Jc of DH asphalt increases, and Jc of KL asphalt decreases and then increases, indicating that the low-temperature performance of SBR-modified asphalt is adversely affected. Because of the addition of PPA, part of the oil with a small molecular weight in the neat asphalt is absorbed. With the increase of PPA dosages, small molecules decrease, the polarity between molecules increases, the motility of large molecules decreases, the asphalt relaxation ability decreases, and the low-temperature crack resistance decreases. The low-temperature performance of PPA-modified asphalt and PPA/SBR-modified asphalt with different dosages was compared and analyzed using Jc. The recommended dosage of PPA is consistent with the results obtained using η_1_.

#### 3.2.5. Relaxation Time (λ)

**Figure 16:**
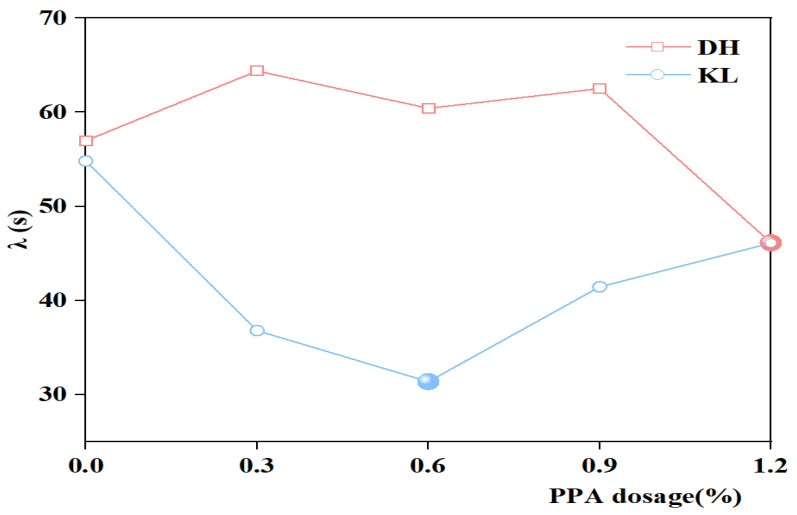
The λ value of single modified asphalt with different PPA dosages.

**Figure 17:**
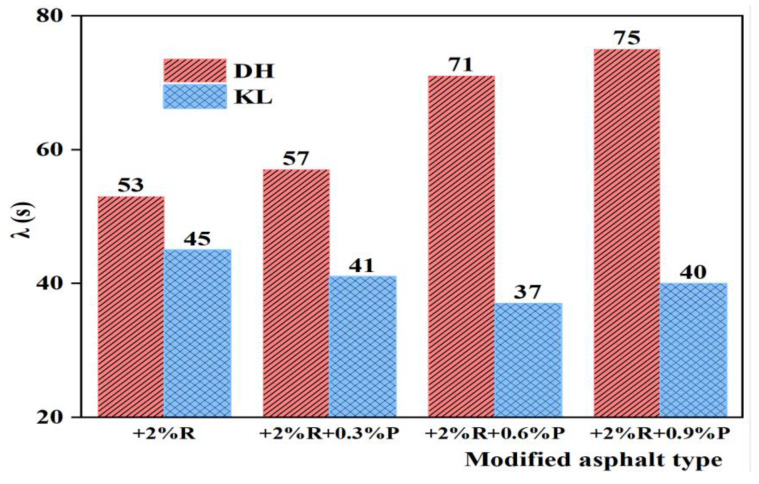
The λ value of composite modified asphalt with different PPA/SBR dosages.

The relaxation time (λ) is an internal time parameter of asphalt. It is calculated from the ratio of the viscous flow parameter η_1_ to the instantaneous modulus of elasticity E_1_, characterizing the stress variation with time. With smaller λ, the asphalt stress relaxation rate is larger, which is more favorable to asphalt stress dissipation, indicating better low-temperature performance. The λ of PPA-modified asphalt and PPA/SBR-modified asphalt is depicted in Figure 16 and Figure 17.

It can be seen from Figure 16 and Figure 17 that for KL neat asphalt, the λ of PPA-modified asphalt and PPA/SBR-modified asphalt is smaller than that of neat asphalt. With the increase of PPA dosages, the λ first decreases and then increases, indicating that the addition of PPA improves both viscosity and elasticity of asphalt. The minimum λ is achieved at a PPA dosage of 0.6%. For DH neat asphalt, the λ of PPA-modified asphalt and PPA/SBR-modified asphalt shows an increasing trend, indicating that the addition of PPA reduces the relaxation rate of asphalt, which is not conducive to asphalt stress dissipation. By analyzing the relationship between the low-temperature performance and relaxation time, the obtained recommended PPA dosages for PPA-modified asphalt and PPA/SBR-modified asphalt are consistent with those obtained using η_1_ and Jc.

Compared to the creep stiffness modulus S and m, K, η_1_, Jc, and λ consider the deformation capacity and stress relaxation capacity of asphalt. They can fit the creep process of low-temperature deformation of asphalt, showing more scientific and reasonable features. Through multi-indicator evaluation and comprehensive analysis of η_1_, Jc, λ, and K, the recommended PPA dosages for PPA-modified asphalt and PPA/SBR-modified asphalt are obtained: DH + 1.2% P, KL + 0.6% P, DH + 2% R + 0.3% P, and KL + 2% R + 0.6% P.

### 3.3. Selection of Low-Temperature Performance Indicators for Asphalt

**Figure 18:**
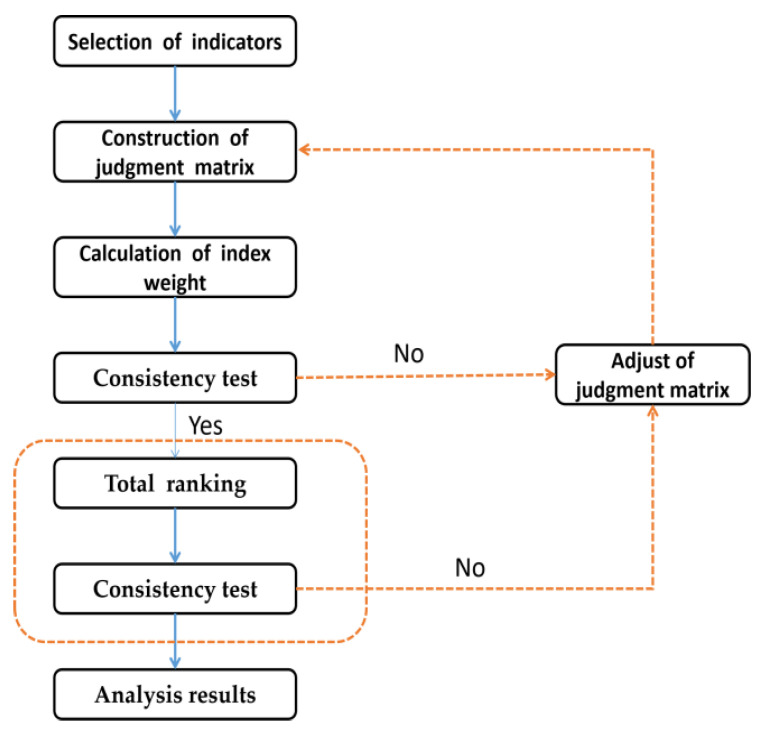
The AHP analysis process.

To evaluate the low-temperature performance of PPA-modified asphalt, the four indicators (K, η_1_, Jc, and λ) were compared and analyzed in terms of instantaneous elasticity, delayed elasticity, and viscosity. In addition, AHP is a comprehensive evaluation method used to solve evaluation problems created by an American operations researcher Saaty [32]. It was used for quantitative analysis to determine the optimal evaluation indicators. The analysis process is shown in Figure 18.

#### 3.3.1. Construction of AHP Hierarchy

**Figure 19:**
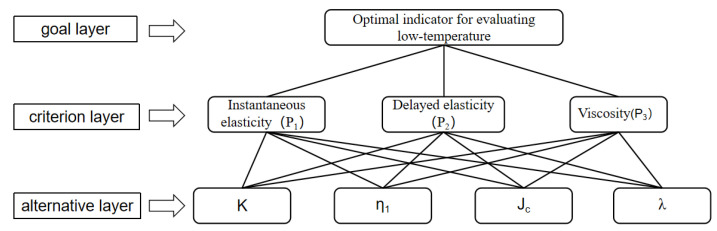
AHP hierarchy.

Create a hierarchical structure model, as shown in Figure 19.

#### 3.3.2. Construction of the Judgment Matrix A for the Goal and Criterion Layers and Consistency Test


$$A=\left[\begin{array}{ccc} 1 & 1/2 & 1/3\\ 2 & 1 & 1/3\\ 3 & 3 & 1 \end{array}\right]$$

Each indicator of matrix A was calculated by $\mathrm{CI}=\overset{3}{\underset{i=1}{\sum }}a_{i}{\mathrm{CI}}_{i}$, $\mathrm{RI}=\overset{3}{\underset{i=1}{\sum }}a_{i}{\mathrm{RI}}_{i}$ and $\mathrm{CR}=\frac{\mathrm{CI}}{\mathrm{RI}}$. The eigenvector W = (0.1593, 0.2519, 0.5889)^T^, the maximum eigenvalue $\lambda_{\max}\;$ = 3.0509, consistency test indicator CI = 0, RI = 0.52, and consistency ratio CR = 0 < 0.1. The consistency test shows that W can be used as a weight vector.

#### 3.3.3. Construction of the Comparison Matrix P_i_ from the Criterion Layer to the Alternative Layer (Comparison Matrix of the i-th Scheme to the Criterion Layer)


$$P_{1}=\left[\begin{array}{cccc} 1 & 2 & 1/3 & 2\\ 1/3 & 1 & 1/2 & 1\\ 3 & 2 & 1 & 3\\ 1/2 & 1 & 1/3 & 1 \end{array}\right]P_{2}=\left[\begin{array}{cccc} 1 & 3 & 1/3 & 3\\ 1/3 & 1 & 1/4 & 1\\ 3 & 4 & 1 & 3\\ 1/3 & 1 & 1/3 & 1 \end{array}\right]\;\;\;\;\;\;P_{3=}\left[\begin{array}{cccc} 1 & 1/3 & 1/4 & 1/5\\ 3 & 1 & 2 & 1/2\\ 4 & 1/2 & 1 & 1\\ 5 & 2 & 1 & 1 \end{array}\right]$$

The comparison matrix P_i_ was analyzed and tested for consistency by the above calculation method of matrix A. The relevant indicators are shown in Table 6. Corresponding eigenvectors of P_i_ are Wp_1_ = (0.2688, 0.1464, 0.4491, 0.1358)^T^, Wp_2_ = (0.2741, 0.1095, 0.4960, 0.1204)^T^, and Wp_3_ = (0.0742, 0.2869, 0.2609, 0.3780)^T^. Consistency test indicators of P_i_ are CIp_1_ = 0.0666, CIp_2_ = 0.4141, and CIp_3_ = 0.0638 with RI = 0.89. The consistency ratios of P_i_ are CRp_1_ = 0.0749 < 0.1, CRp_2_ = 0.0465 < 0.1, and CRp_3_ = 0.0659 < 0.1. The CR values of the above comparison matrix P_i_ are all smaller than 0.1, consistent with the requirements of the consistency test. Therefore, P_i_ can be used as the comparison matrix of the criterion layer to the scheme layer.

#### 3.3.4. Total AHP Ranking and the Consistency Test

Based on the data obtained from the first two steps, the total ranking of AHP is listed in Table 7.

The consistency test was performed for the total AHP ranking, with CI = 0.0586 and CR = 0.0659 < 0.1, consistent with the requirements. In addition, the evaluation indicators are quantitatively analyzed in terms of instantaneous elasticity, delayed elasticity, and viscosity. The results show that the weight value from large to small is Jc > λ > η_1_ > K. Therefore, it is recommended to use the Jc index to evaluate the low-temperature performance of PPA-modified asphalt. However, the calculation of the Jc index is complex and requires high accuracy, which is suitable for laboratory research on the low-temperature performance of PPA-modified asphalt. The weight value of the λ index is also relatively large, with a simple calculation and a clear physical meaning, which is suitable for engineering applications to evaluate the low-temperature performance of PPA-modified asphalt.

## 4. Conclusions

The present study investigated the low-temperature performance and evaluation indicators of PPA-modified asphalt and PPA/SBR-modified asphalt and explored the mechanism of PPA modification. Relevant low-temperature performance indicators were selected based on low-temperature deformation and stress relaxation of modified asphalt. Then, these indicators were optimized by AHP. The conclusions are drawn as follows:

(1) Based on SEM images: After the addition of PPA, the particle size of the asphalt surface and agglomeration degree of SBR-modified asphalt were reduced, indicating that PPA can effectively improve the compatibility of SBR and neat asphalt.
(2) Based on the FTIR test: The infrared spectrum of PPA-modified asphalt showed the disappearance and generation of absorption peaks at 1725 cm^−1^–1480 cm^−1^, 2358 cm^−1^, and 2387 cm^−1^, which indicated that the chemical reaction occurred when PPA was added into the neat asphalt, and phosphate ester was formed. In the infrared spectra of PPA/SBR modified asphalt, the characteristic peaks disappear and produce at 1000 cm^−1^–1600 cm^−1^ and 1600 cm^−1^, indicating that the composite modification was mainly chemical.
(3) Based on the BBR test: The low-temperature performance of PPA-modified asphalt was evaluated comprehensively by the Burgers model. The low-temperature performance of asphalt with different PPA dosages evaluated only by creep stiffness modulus (S) or creep rate (m) leads to different trends, which cannot accurately evaluate the low-temperature performance of modified asphalt. Additionally, a good correlation can be obtained by fitting the Burgers model with the low-temperature creep flexibility. Considering the deformation and stress relaxation of asphalt, the K index and the Burgers model-based η_1_, Jc, and λ were selected to evaluate the low-temperature performance of asphalt. The multi-indicator analysis shows that the recommended PPA dosage is 1.2% for DH asphalt, 0.6% for KL asphalt, 0.3% for DH-PPA/SBR-modified asphalt, and 0.6% for KL-PPA/SBR-modified asphalt.
(4) The indicators η_1_, Jc, λ, and K were analyzed by AHP. The results showed that Jc has the highest weight value of 0.3501, with a heavy calculation burden. It is recommended to use this indicator in the research work for evaluating the low-temperature performance of PPA-modified asphalt. The weight value of λ (0.2745) is also large but simple to calculate and can be used in practical engineering.

This research comprehensively analyzed the low-temperature performance evaluation indicators of PPA-modified asphalt. The selection of these indicators was recommended in terms of laboratory research and engineering practice, providing a theoretical basis for the application of the PPA modifier. To better fit the actual situation of the project, it is necessary to further study the low-temperature performance evaluation indicators of PPA-modified asphalt mixture and establish a low-temperature prediction model between PPA-modified asphalt and PPA-modified asphalt mixture.

## Figures and Tables

**Table 1:** Technical properties of asphalt.

Test Items		Unit	Test Results		Test Methods (JTG E20-2011)
			DH	KL	
Softening point (R&B)		°C	53.1	49.5	T 0606
Ductility (50 mm/min, 10 °C)		cm	39.7	>100	T 0605
Needle penetration (25 °C, 5 s, 100 g)		0.1 mm	64.2	93.5	T 0604
Solubility		%	99.9	99.9	T 0607
Density (15 °C)		g/cm^3^	1.033	1.031	T 0603
Flash point		°C	279	283	T 0611
RTFOT * Residues	Mass change	%	0.47	0.58	T 0610
	Residual needle penetration rate (25 °C)	%	70.2	64.3	T 0604
	Residual ductility (10 °C)	cm	14.1	9.5	T 0605

* Rolling Thin Film Oven Test.

**Table 2:** Main technical specifications of SBR.

Technical Specifications	Unit	Measured Value
Granularity	Mesh	10~80
Combined styrene content	%	10~50
Mooney viscosity	ML	45~65
tensile stress at 300% elongation	MPa	15
Tensile strength	MPa	25

**Table 3:** Basic indicators of PPA.

PPA Types	Test Indicators			
	P_2_O_5_ (Mass Fraction) (%)	Chloride (Cl, %)	Heavy Metals (Pb, %)	Iron (Fe, %)
PPA-110	82.0	≤0.001	≤0.003	≤0.002

**Table 4:** BBR test results of PPA-modified asphalt.

PPA Dosage (%)	BBR Indicators	Test Data Corresponding to Different Neat Asphalts	
		DH	KL
0	S (MPa)	105	87.2
	m	0.458	0.515
0.3	S (MPa)	98.1	69.3
	m	0.431	0.526
0.6	S (MPa)	96.6	67.6
	m	0.436	0.542
0.9	S (MPa)	85.4	68.4
	m	0.440	0.465
1.2	S (MPa)	87.3	71.1
	m	0.448	0.487

**Table 5:** BBR test results of PPA/SBR-modified asphalt.

Asphalt Specimens	BBR Indicators	Test Data Corresponding to Different Neat Asphalts	
		DH	KL
+2% R	S (MPa)	118	88.9
	m	0.423	0.417
+2% R + 0.3% P	S (MPa)	91	77.9
	m	0.417	0.501
+2% R + 0.6% P	S (MPa)	109	57.9
	m	0.407	0.522
+2% R + 0.9% P	S (MPa)	106	68.3
	m	0.404	0.510

**Table 6:** Consistency test indicators of the comparison matrix P_i_.

Consistency Test Indicators	P_1_	P_2_	P_3_
Wp_i_	0.2688	0.2741	0.0742
	0.1464	0.1095	0.2869
	0.4491	0.4960	0.2609
	0.1358	0.1204	0.3780
CI	0.0666	0.0414	0.0638
RI	0.89	0.89	0.89
CR	0.0749	0.0465	0.0659

**Table 7:** Total AHP ranking.

Indicators	P_1_	P_2_	P_3_	Total Weight
K	0.2688	0.2741	0.0742	0.1555
*η*_1_	0.1464	0.1095	0.2869	0.2198
*J*_c_	0.4491	0.4960	0.2609	0.3501
*λ*	0.1358	0.1204	0.3780	0.2745

## Data Availability

Data is contained within the article.

## References

[1] Porto M., Caputo P., Loise V., Eskandarsefat S., Teltayev B., Oliviero Rossi C. (2019). Bitumen and Bitumen Modification: A Review on Latest Advances. Appl. Sci..

[2] Han Y., Cui B., Tian J., Ding J., Ni F., Lu D. (2022). Evaluating the effects of styrene-butadiene rubber (SBR) and polyphosphoric acid (PPA) on asphalt adhesion performance. Constr. Build. Mater..

[3] Han Y., Ding J., Han D., Zhao Z., Ma X., Ni F. (2022). Evaluating the thermal aging-induced raveling potential of thin friction course (TFC). Constr. Build. Mater..

[4] Zhang F., Yu J. (2010). The research for high-performance SBR compound modified asphalt. Constr. Build. Mater..

[5] Li X., Pei J., Shen J., Li R. (2019). Experimental Study on the High-Temperature and Low-Temperature Performance of Polyphosphoric Acid/Styrene-Butadiene-Styrene Composite-Modified Asphalt. Adv. Mater. Sci. Eng..

[6] Fu G. (2017). Research on Modification Mechanisms of Polyphosphoric Acid on Asphalt and the Performance of Polyphosphoric Acid Modified Asphalt Mixture. Master’s Thesis.

[7] Wang L., Wang Z., Li C. (2017). High temperature rheological properties of polyphosphoric acid modifier asphalt. Acta Mater. Compos. Sin..

[8] Sarnowski M. (2015). Rheological properties of road bitumen binders modified with SBS polymer and polyphosphoric acid. Roads Bridg.-Drog. I Mosty.

[9] Liu Z., Li S., Wang Y. (2022). Waste engine oil and polyphosphoric acid enhanced the sustainable self-healing of asphalt binder and its fatigue behavior. J. Clean. Prod..

[10] Wei J., Shi S., Zhou Y., Li P., Chen Z., Guan Y. (2019). Rheological property of polyphosphoric acid modified asphalt. J. Tranffic Transp. Eng..

[11] Fu G., Zhao Y., Sun Q. (2017). Modification mechanisms of polyphosphoric acid and SBS on asphalt. Acta Mater. Compos. Sinaca.

[12] Song R., Sha A., Shi K., Li J., Li X., Zhang F. (2021). Polyphosphoric acid and plasticizer modified asphalt: Rheological properties and modification mechanism. Constr. Build. Mater..

[13] Baldino N., Gabriele D., Rossi C.O., Oliviero C., Seta L., Lupi F.R., Caputo P. (2012). Low temperature rheology of polyphosphoric acid (PPA) added bitumen. Constr. Build. Mater..

[14] Zhou Y., Huang D., Fu X. (2017). Low-Temperature performance of polyphosphoric acid composite modified asphalt. J. Build. Mater..

[15] Aflaki S., Hajikarimi P., Fini E.H., Zada B. (2014). Comparing Effects of Biobinder with Other Asphalt Modifiers on Low-Temperature Characteristics of Asphalt. J. Mater. Civ. Eng..

[16] Zhang L., Huang W., Wei M., Ouyang X., Wang Y. (2020). Analysis of rheological properties of polyphosphoric acid modified asphalt. J. Mater. Sci. Eng..

[17] Liu H., Zhang M., Huang L., Chang R., Hao P. (2016). Rheological and anti-aging propertied of polyphosphoric acid composite styrene butadiene styrene modified asphalt. J. Southeast Univ..

[18] Baldino N., Gabriele D., Lupi F.R., Rossi C.O., Caputo P., Falvo T. (2013). Rheological effects on bitumen of polyphosphoric acid (PPA) addition. Constr. Build. Mater..

[19] Wang L., Wang Z., Li C. (2017). Low temperature performance of polyphosphoric acid asphalt and polyphosphoric acid-SBS modifier asphalt. Acta Mater. Compos. Sin..

[20] Thomas K.P., Turner T.F. (2008). Polyphosphoric-acid modification of asphalt binders: Impact on rheological and thermal properties. Road Mater. Pavement Des..

[21] Edwards Y., Tasdemir Y., Isacsson U. (2007). Rheological effects of commercial waxes and polyphosphoric acid in bitumen 160/220—High and medium temperature performance. Constr. Build. Mater..

[22] Yin Y., Zhang X., Zou G. (2010). Investigation into Low-temperature performance of asphalt mixtures based on glass transition temperature. J. South China Univ. Technol..

[23] Huang W., Fu X., Li Y., Liu S. (2017). Evaluation of low temperature performance and correlation analysis on low temperature indexes of SBS modified asphalts. J. Build. Mater..

[24] Huang Q., Liu A., Yan E. (2022). Multi-index evaluation and study of low temperature performance of high modified asphalt. Highway.

[25] Zhou Y., Wei J., Shi S., Gao J., Duan X., Chen Y. (2018). Properties of composite-modified asphalt with polyphosphoric acid and rubber powder. J. Chang. Univ..

[26] Zhang M. (2012). Research on the Microstructure and Technical Performance of Polyphosphoric Acid Modified Asphalt. Ph.D. Thesis.

[27] (2012). Standard Method of Test for Determining the Flexural Creep Stiffness of Asphalt Binder Using the Bending Beam Rheometer (BBR).

[28] Tan Y., Fu Y., Ji L., Meng D. (2016). Low-temperature evaluation index of rubber asphalt. J. Harbin Inst. Technol..

[29] Wang W., Luo R., Feng G., Wang L.G. (2016). Impact factors in rotational viscosity tests. J. Wuhan Univ. Technol..

[30] Wang K., Hao P. (2016). Analysis of asphalt low temperature performance and viscoelasticity based on BBR test. J. Liaoning Tech. Univ..

[31] Dong Y., Tan Y., Liu H., Li Z. (2015). Development and application of fibre grating inclinometer for monitoring the rock and soil slope deformation. Highway.

[32] Saaty T.L. (1990). How to make a decision: The analytic hierarchy process. Eur. J. Oper. Res..

